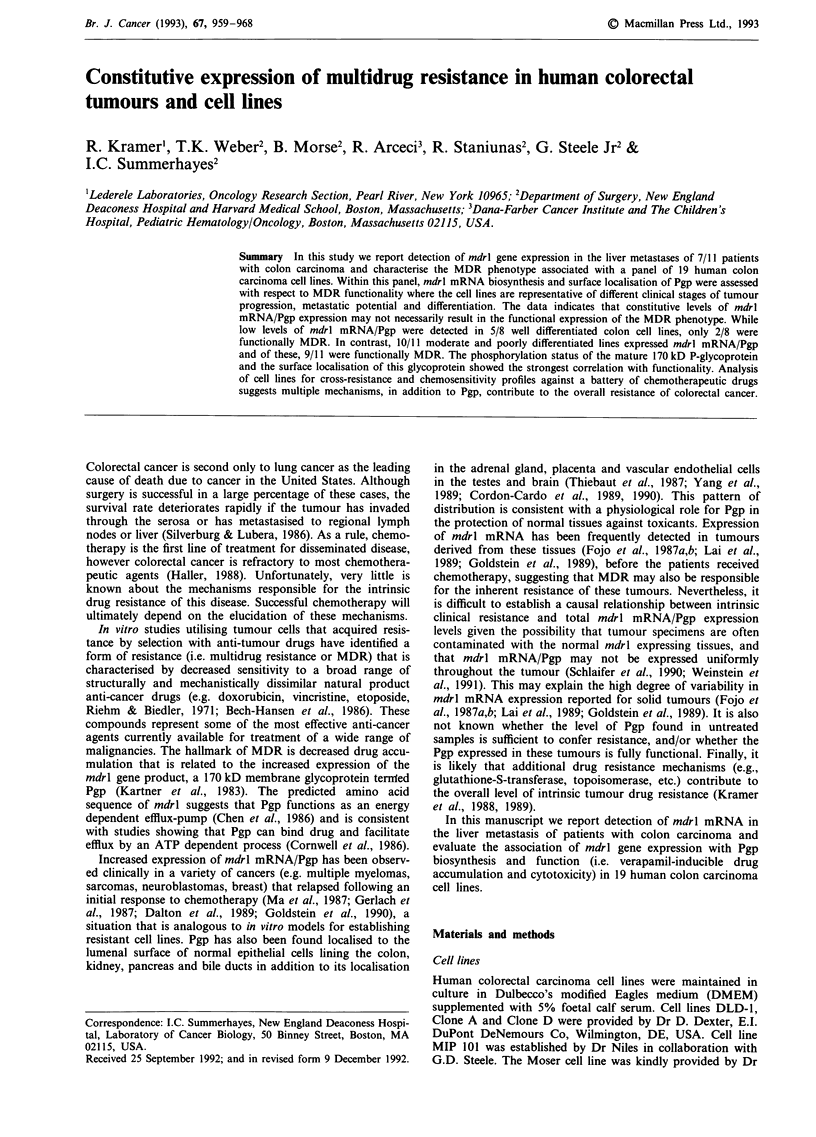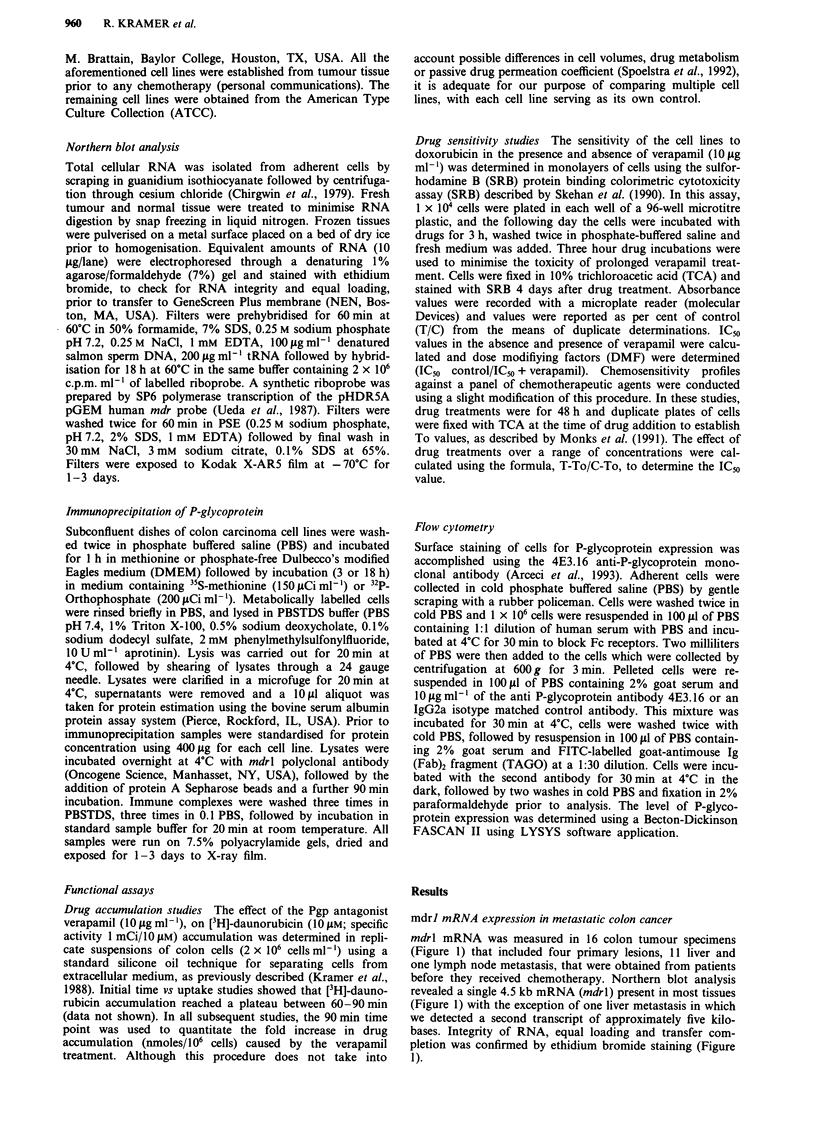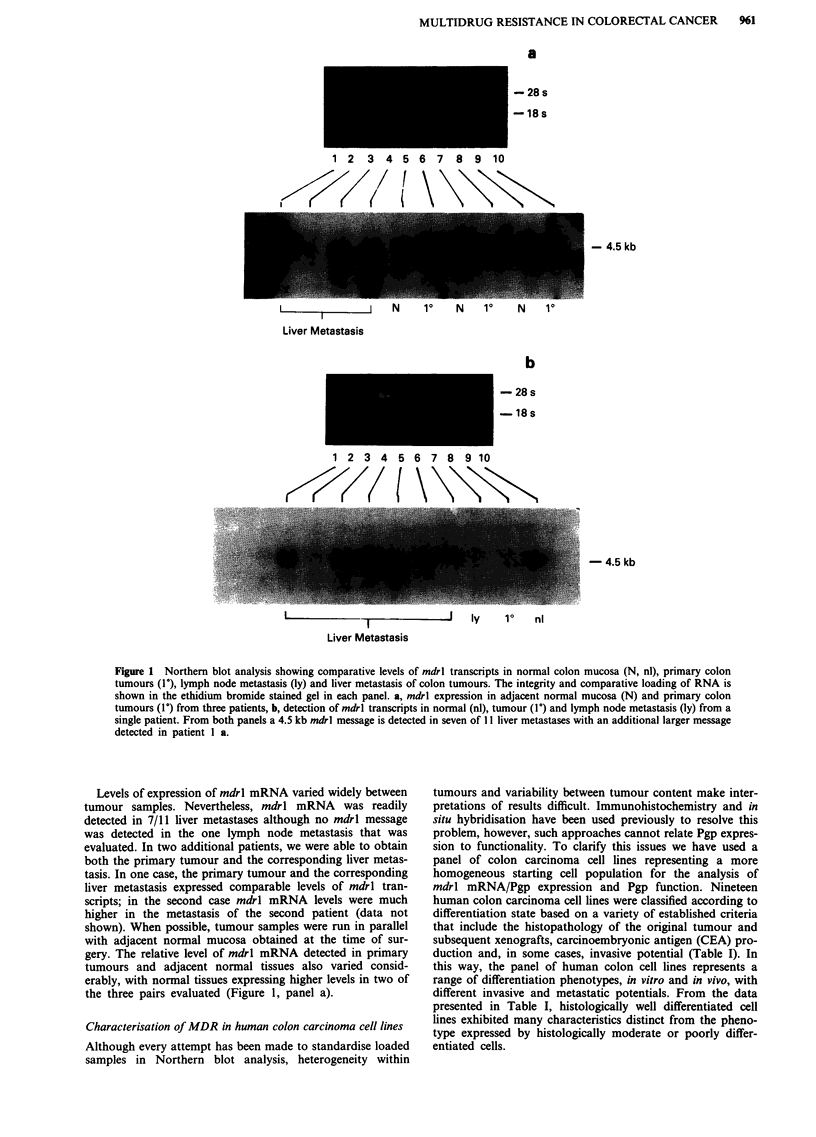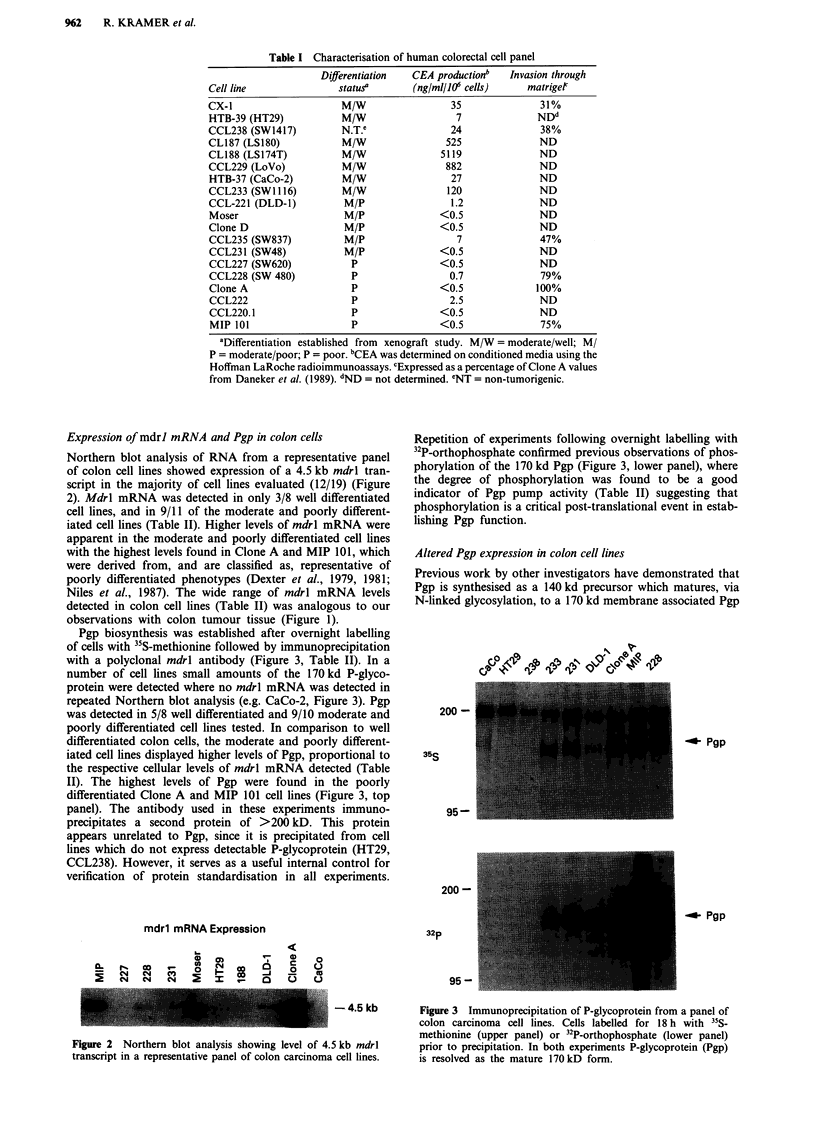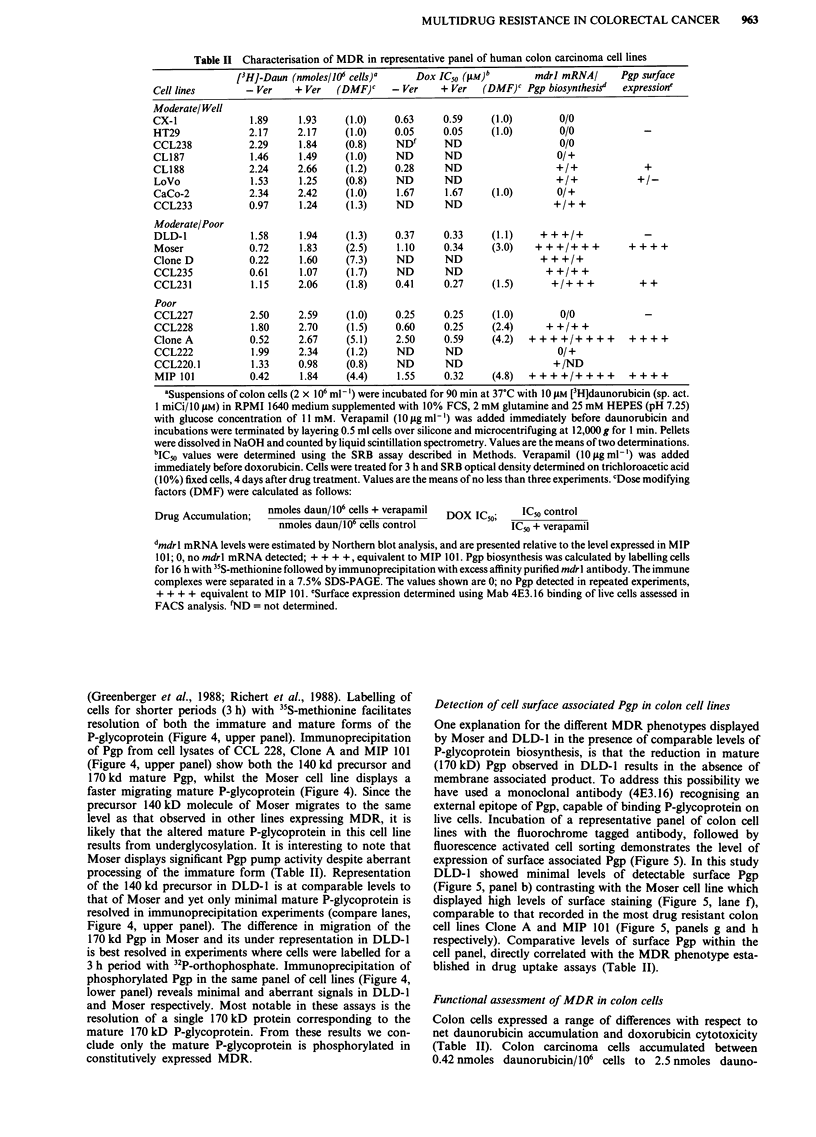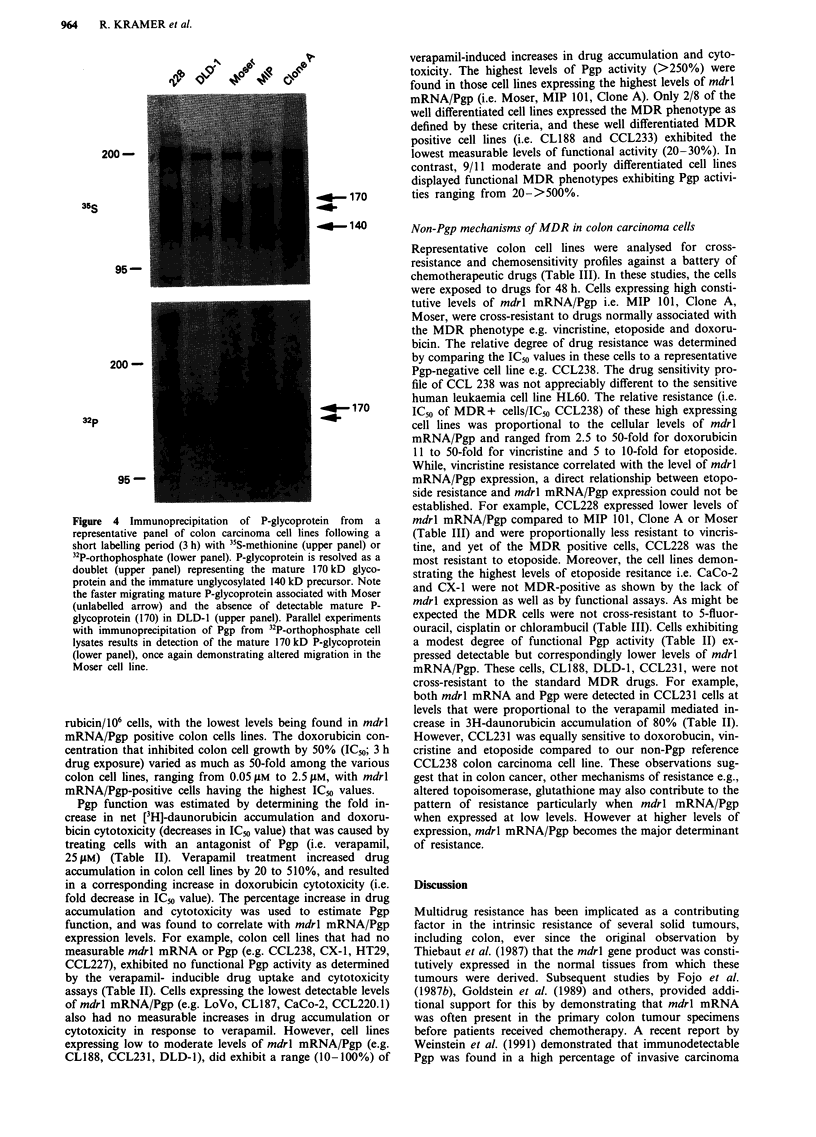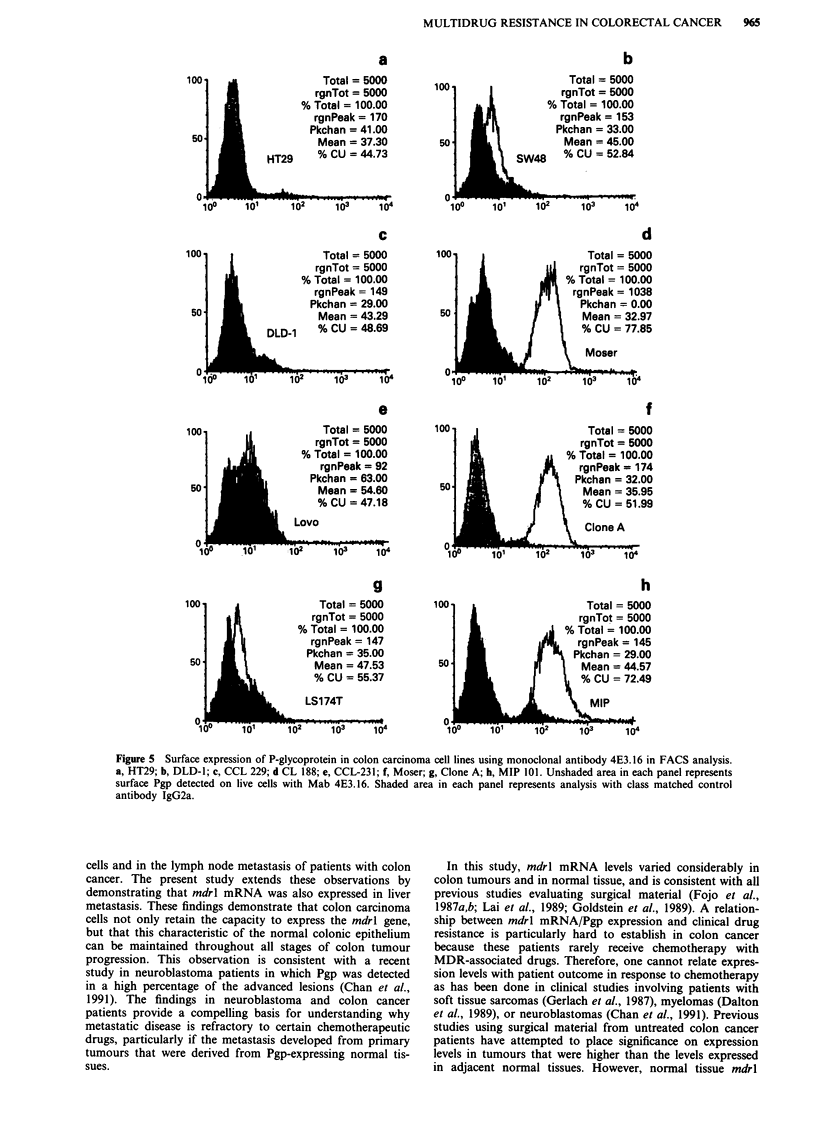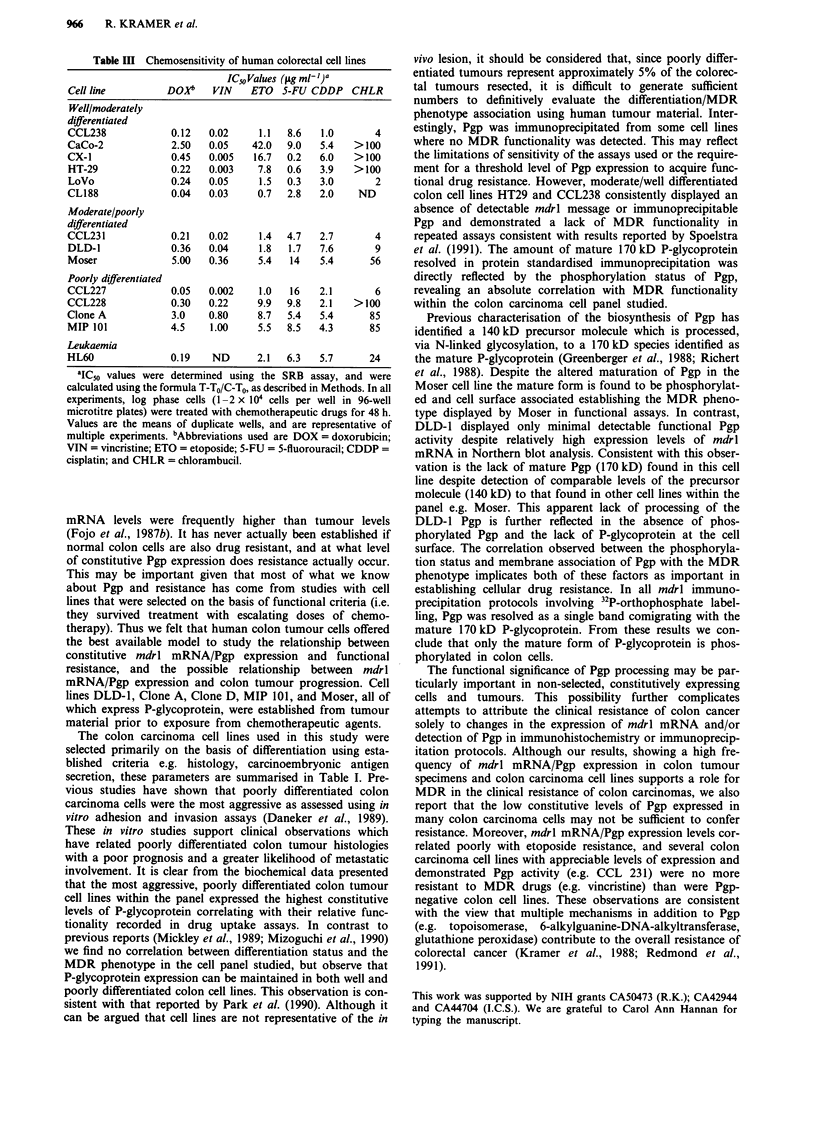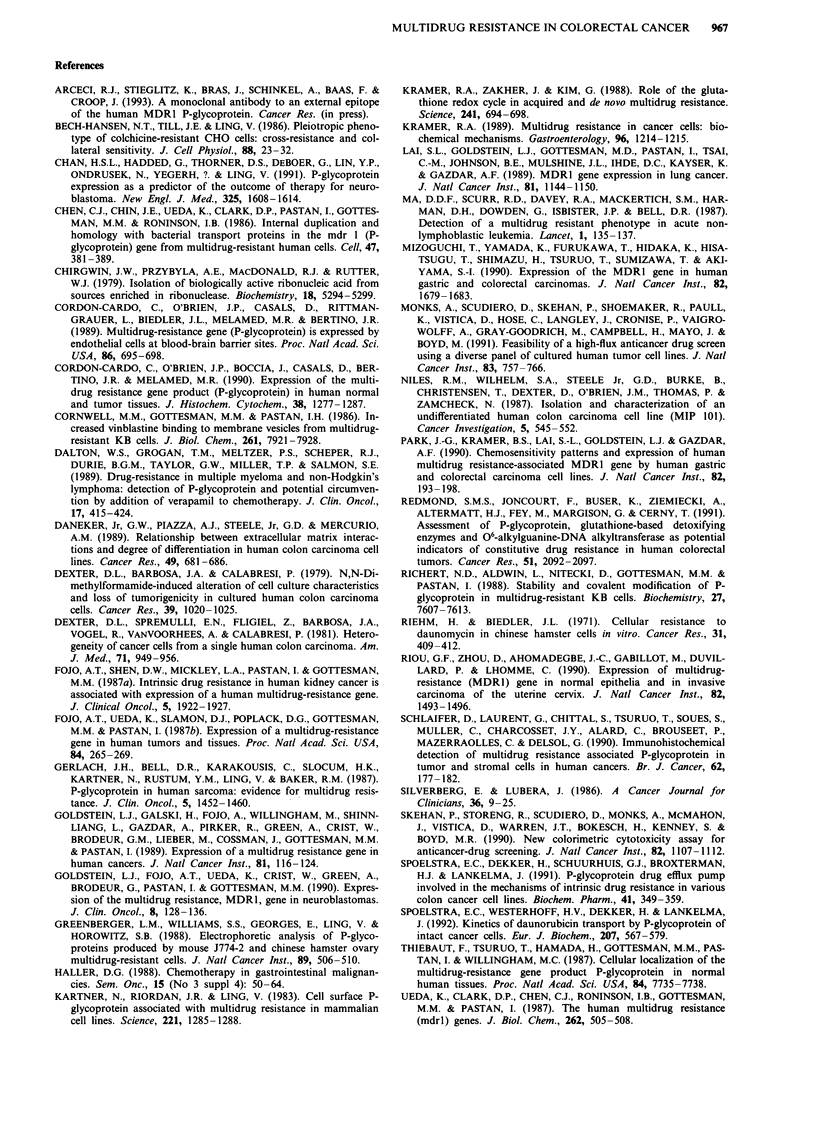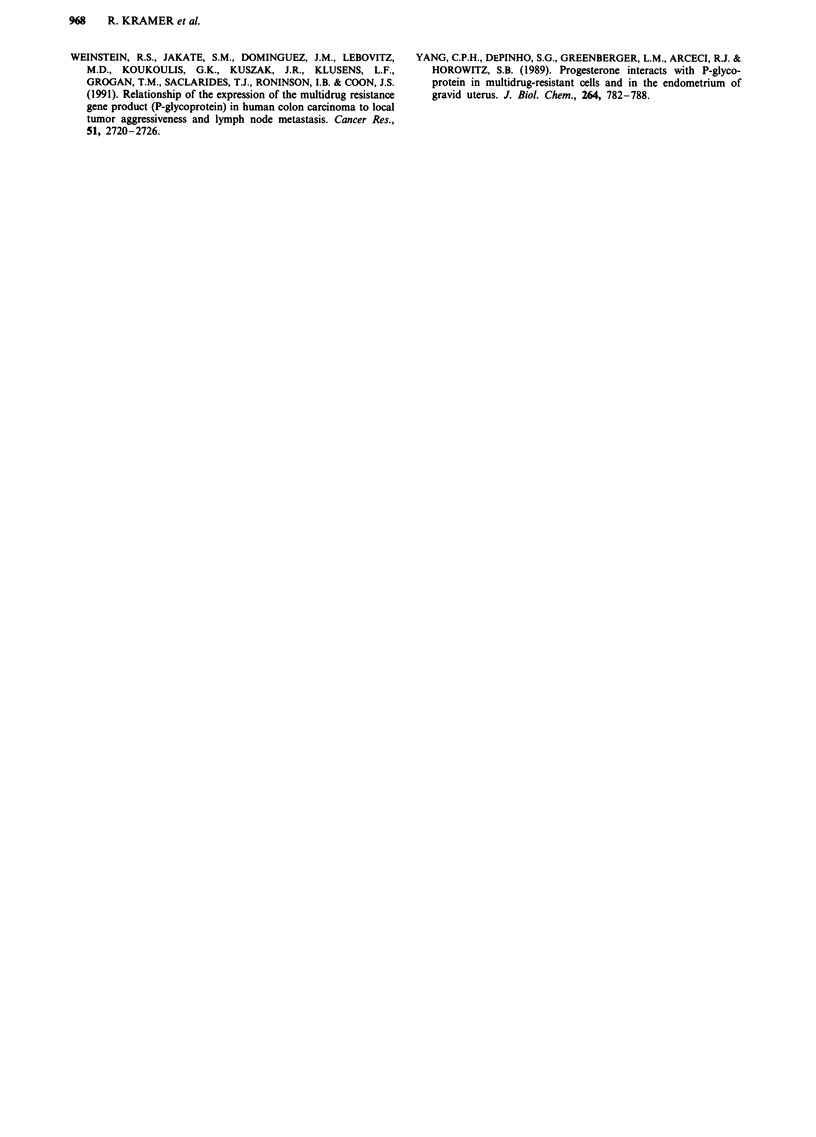# Constitutive expression of multidrug resistance in human colorectal tumours and cell lines.

**DOI:** 10.1038/bjc.1993.177

**Published:** 1993-05

**Authors:** R. Kramer, T. K. Weber, B. Morse, R. Arceci, R. Staniunas, G. Steele, I. C. Summerhayes

**Affiliations:** Lederele Laboratories, Oncology Research Section, Pearl River, New York 10965.

## Abstract

**Images:**


Br. J. Cancer (1993), 67, 959–968 © Macmillan Press Ltd., 1993

### Constitutive expression of multidrug resistance in human colorectal tumours and cell lines

R. Kramer[1], T.K. Weber[2], B. Morse[2], R. Arceci[3], R. Staniunas[2], G. Steele Jr[2] & I.C. Summerhayes[2]

[1]*Lederele Laboratories, Oncology Research Section, Pearl River, New York 10965;* [2]*Department of Surgery, New England Deaconess Hospital and Harvard Medical School, Boston, Massachusetts;* [3]*Dana-Farber Cancer Institute and The Children's Hospital, Pediatric Hematology/Oncology, Boston, Massachusetts 02115, USA.*

**Summary** In this study we report detection of *mdr*1 gene expression in the liver metastases of 7/11 patients with colon carcinoma and characterise the MDR phenotype associated with a panel of 19 human colon carcinoma cell lines. Within this panel, *mdr*1 mRNA biosynthesis and surface localisation of Pgp were assessed with respect to MDR functionality where the cell lines are representative of different clinical stages of tumour progression, metastatic potential and differentiation. The data indicates that constitutive levels of *mdr*1 mRNA/Pgp expression may not necessarily result in the functional expression of the MDR phenotype. While low levels of *mdr*1 mRNA/Pgp were detected in 5/8 well differentiated colon cell lines, only 2/8 were functionally MDR. In contrast, 10/11 moderate and poorly differentiated lines expressed *mdr*1 mRNA/Pgp and of these, 9/11 were functionally MDR. The phosphorylation status of the mature 170 kD P-glycoprotein and the surface localisation of this glycoprotein showed the strongest correlation with functionality. Analysis of cell lines for cross-resistance and chemosensitivity profiles against a battery of chemotherapeutic drugs suggests multiple mechanisms, in addition to Pgp, contribute to the overall resistance of colorectal cancer.

Colorectal cancer is second only to lung cancer as the leading cause of death due to cancer in the United States. Although surgery is successful in a large percentage of these cases, the survival rate deteriorates rapidly if the tumour has invaded through the serosa or has metastasised to regional lymph nodes or liver (Silverburg & Lubera, 1986). As a rule, chemotherapy is the first line of treatment for disseminated disease, however colorectal cancer is refractory to most chemotherapeutic agents (Haller, 1988). Unfortunately, very little is known about the mechanisms responsible for the intrinsic drug resistance of this disease. Successful chemotherapy will ultimately depend on the elucidation of these mechanisms.

*In vitro* studies utilising tumour cells that acquired resistance by selection with anti-tumour drugs have identified a form of resistance (i.e. multidrug resistance or MDR) that is characterised by decreased sensitivity to a broad range of structurally and mechanistically dissimilar natural product anti-cancer drugs (e.g. doxorubicin, vincristine, etoposide, Riehm & Biedler, 1971; Bech-Hansen *et al*., 1986). These compounds represent some of the most effective anti-cancer agents currently available for treatment of a wide range of malignancies. The hallmark of MDR is decreased drug accumulation that is related to the increased expression of the *mdr*1 gene product, a 170 kD membrane glycoprotein termed Pgp (Kartner *et al*., 1983). The predicted amino acid sequence of *mdr*1 suggests that Pgp functions as an energy dependent efflux-pump (Chen *et al*., 1986) and is consistent with studies showing that Pgp can bind drug and facilitate efflux by an ATP dependent process (Cornwell *et al*., 1986).

Increased expression of *mdr*1 mRNA/Pgp has been observed clinically in a variety of cancers (e.g. multiple myelomas, sarcomas, neuroblastomas, breast) that relapsed following an initial response to chemotherapy (Ma *et al*., 1987; Gerlach *et al*., 1987; Dalton *et al*., 1989; Goldstein *et al*., 1990), a situation that is analogous to *in vitro* models for establishing resistant cell lines. Pgp has also been found localised to the lumenal surface of normal epithelial cells lining the colon, kidney, pancreas and bile ducts in addition to its localisation in the adrenal gland, placenta and vascular endothelial cells in the testes and brain (Thiebaut *et al*., 1987; Yang *et al*., 1989; Cordon-Cardo *et al*., 1989, 1990). This pattern of distribution is consistent with a physiological role for Pgp in the protection of normal tissues against toxicants. Expression of *mdr*1 mRNA has been frequently detected in tumours derived from these tissues (Fojo *et al*., 1987*a*,*b*; Lai *et al*., 1989; Goldstein *et al*., 1989), before the patients received chemotherapy, suggesting that MDR may also be responsible for the inherent resistance of these tumours. Nevertheless, it is difficult to establish a causal relationship between intrinsic clinical resistance and total *mdr*1 mRNA/Pgp expression levels given the possibility that tumour specimens are often contaminated with the normal *mdr*1 expressing tissues, and that *mdr*1 mRNA/Pgp may not be expressed uniformly throughout the tumour (Schlaifer *et al*., 1990; Weinstein *et al*., 1991). This may explain the high degree of variability in *mdr*1 mRNA expression reported for solid tumours (Fojo *et al*., 1987*a*,*b*; Lai *et al*., 1989; Goldstein *et al*., 1989). It is also not known whether the level of Pgp found in untreated samples is sufficient to confer resistance, and/or whether the Pgp expressed in these tumours is fully functional. Finally, it is likely that additional drug resistance mechanisms (e.g., glutathione-S-transferase, topoisomerase, etc.) contribute to the overall level of intrinsic tumour drug resistance (Kramer *et al*., 1988, 1989).

In this manuscript we report detection of *mdr*1 mRNA in the liver metastasis of patients with colon carcinoma and evaluate the association of *mdr*1 gene expression with Pgp biosynthesis and function (i.e. verapamil-inducible drug accumulation and cytotoxicity) in 19 human colon carcinoma cell lines.

### Materials and methods

### *Cell lines*

Human colorectal carcinoma cell lines were maintained in culture in Dulbecco's modified Eagles medium (DMEM) supplemented with 5% foetal calf serum. Cell lines DLD-1, Clone A and Clone D were provided by Dr D. Dexter, E.I. DuPont DeNemours Co, Wilmington, DE, USA. Cell line MIP 101 was established by Dr Niles in collaboration with G.D. Steele. The Moser cell line was kindly provided by Dr

Correspondence: I.C. Summerhayes, New England Deaconess Hospital, Laboratory of Cancer Biology, 50 Binney Street, Boston, MA 02115, USA.
Received 25 September 1992; and in revised form 9 December 1992.

M. Brattain, Baylor College, Houston, TX, USA. All the aforementioned cell lines were established from tumour tissue prior to any chemotherapy (personal communications). The remaining cell lines were obtained from the American Type Culture Collection (ATCC).

*Northern blot analysis*

Total cellular RNA was isolated from adherent cells by scraping in guanidium isothiocyanate followed by centrifugation through cesium chloride (Chirgwin et al., 1979). Fresh tumour and normal tissue were treated to minimise RNA digestion by snap freezing in liquid nitrogen. Frozen tissues were pulverised on a metal surface placed on a bed of dry ice prior to homogenisation. Equivalent amounts of RNA (10 μg/lane) were electrophoresed through a denaturing 1% agarose/formaldehyde (7%) gel and stained with ethidium bromide, to check for RNA integrity and equal loading, prior to transfer to GeneScreen Plus membrane (NEN, Boston, MA, USA). Filters were prehybridised for 60 min at 60°C in 50% formamide, 7% SDS, 0.25 M sodium phosphate pH 7.2, 0.25 M NaCl, 1 mM EDTA, 100 μg $ml^{-1}$ denatured salmon sperm DNA, 200 μg $ml^{-1}$ tRNA followed by hybridisation for 18 h at 60°C in the same buffer containing $2 \times 10^6$ c.p.m. $ml^{-1}$ of labelled riboprobe. A synthetic riboprobe was prepared by SP6 polymerase transcription of the pHDR5A pGEM human *mdr* probe (Ueda et al., 1987). Filters were washed twice for 60 min in PSE (0.25 M sodium phosphate, pH 7.2, 2% SDS, 1 mM EDTA) followed by final wash in 30 mM NaCl, 3 mM sodium citrate, 0.1% SDS at 65%. Filters were exposed to Kodak X-AR5 film at −70°C for 1–3 days.

*Immunoprecipitation of P-glycoprotein*

Subconfluent dishes of colon carcinoma cell lines were washed twice in phosphate buffered saline (PBS) and incubated for 1 h in methionine or phosphate-free Dulbecco's modified Eagles medium (DMEM) followed by incubation (3 or 18 h) in medium containing $^{35}$S-methionine (150 μCi $ml^{-1}$) or $^{32}$P-Orthophosphate (200 μCi $ml^{-1}$). Metabolically labelled cells were rinsed briefly in PBS, and lysed in PBSTDS buffer (PBS pH 7.4, 1% Triton X-100, 0.5% sodium deoxycholate, 0.1% sodium dodecyl sulfate, 2 mM phenylmethylsulfonylfluoride, 10 U $ml^{-1}$ aprotinin). Lysis was carried out for 20 min at 4°C, followed by shearing of lysates through a 24 gauge needle. Lysates were clarified in a microfuge for 20 min at 4°C, supernatants were removed and a 10 μl aliquot was taken for protein estimation using the bovine serum albumin protein assay system (Pierce, Rockford, IL, USA). Prior to immunoprecipitation samples were standardised for protein concentration using 400 μg for each cell line. Lysates were incubated overnight at 4°C with *mdr*1 polyclonal antibody (Oncogene Science, Manhasset, NY, USA), followed by the addition of protein A Sepharose beads and a further 90 min incubation. Immune complexes were washed three times in PBSTDS, three times in 0.1 PBS, followed by incubation in standard sample buffer for 20 min at room temperature. All samples were run on 7.5% polyacrylamide gels, dried and exposed for 1–3 days to X-ray film.

*Functional assays*

*Drug accumulation studies* The effect of the Pgp antagonist verapamil (10 μg $ml^{-1}$), on [$^3$H]-daunorubicin (10 μM; specific activity 1 mCi/10 μM) accumulation was determined in replicate suspensions of colon cells ($2 \times 10^6$ cells $ml^{-1}$) using a standard silicone oil technique for separating cells from extracellular medium, as previously described (Kramer et al., 1988). Initial time *vs* uptake studies showed that [$^3$H]-daunorubicin accumulation reached a plateau between 60–90 min (data not shown). In all subsequent studies, the 90 min time point was used to quantitate the fold increase in drug accumulation (nmoles/$10^6$ cells) caused by the verapamil treatment. Although this procedure does not take into account possible differences in cell volumes, drug metabolism or passive drug permeation coefficient (Spoelstra et al., 1992), it is adequate for our purpose of comparing multiple cell lines, with each cell line serving as its own control.

*Drug sensitivity studies* The sensitivity of the cell lines to doxorubicin in the presence and absence of verapamil (10 μg $ml^{-1}$) was determined in monolayers of cells using the sulforhodamine B (SRB) protein binding colorimetric cytotoxicity assay (SRB) described by Skehan et al. (1990). In this assay, $1 \times 10^4$ cells were plated in each well of a 96-well microtitre plastic, and the following day the cells were incubated with drugs for 3 h, washed twice in phosphate-buffered saline and fresh medium was added. Three hour drug incubations were used to minimise the toxicity of prolonged verapamil treatment. Cells were fixed in 10% trichloroacetic acid (TCA) and stained with SRB 4 days after drug treatment. Absorbance values were recorded with a microplate reader (molecular Devices) and values were reported as per cent of control (T/C) from the means of duplicate determinations. $IC_{50}$ values in the absence and presence of verapamil were calculated and dose modifiying factors (DMF) were determined ($IC_{50}$ control/$IC_{50}$ + verapamil). Chemosensitivity profiles against a panel of chemotherapeutic agents were conducted using a slight modification of this procedure. In these studies, drug treatments were for 48 h and duplicate plates of cells were fixed with TCA at the time of drug addition to establish To values, as described by Monks et al. (1991). The effect of drug treatments over a range of concentrations were calculated using the formula, T-To/C-To, to determine the $IC_{50}$ value.

*Flow cytometry*

Surface staining of cells for P-glycoprotein expression was accomplished using the 4E3.16 anti-P-glycoprotein monoclonal antibody (Arceci et al., 1993). Adherent cells were collected in cold phosphate buffered saline (PBS) by gentle scraping with a rubber policeman. Cells were washed twice in cold PBS and $1 \times 10^6$ cells were resuspended in 100 μl of PBS containing 1:1 dilution of human serum with PBS and incubated at 4°C for 30 min to block Fc receptors. Two milliliters of PBS were then added to the cells which were collected by centrifugation at 600 *g* for 3 min. Pelleted cells were resuspended in 100 μl of PBS containing 2% goat serum and 10 μg $ml^{-1}$ of the anti P-glycoprotein antibody 4E3.16 or an IgG2a isotype matched control antibody. This mixture was incubated for 30 min at 4°C, cells were washed twice with cold PBS, followed by resuspension in 100 μl of PBS containing 2% goat serum and FITC-labelled goat-antimouse Ig $(Fab)_2$ fragment (TAGO) at a 1:30 dilution. Cells were incubated with the second antibody for 30 min at 4°C in the dark, followed by two washes in cold PBS and fixation in 2% paraformaldehyde prior to analysis. The level of P-glycoprotein expression was determined using a Becton-Dickinson FASCAN II using LYSYS software application.

### Results

*mdr1 mRNA expression in metastatic colon cancer*

*mdr*1 mRNA was measured in 16 colon tumour specimens (Figure 1) that included four primary lesions, 11 liver and one lymph node metastasis, that were obtained from patients before they received chemotherapy. Northern blot analysis revealed a single 4.5 kb mRNA (*mdr*1) present in most tissues (Figure 1) with the exception of one liver metastasis in which we detected a second transcript of approximately five kilobases. Integrity of RNA, equal loading and transfer completion was confirmed by ethidium bromide staining (Figure 1).

**Figure 1** Northern blot analysis showing comparative levels of *mdr*1 transcripts in normal colon mucosa (N, nl), primary colon tumours (1°), lymph node metastasis (ly) and liver metastasis of colon tumours. The integrity and comparative loading of RNA is shown in the ethidium bromide stained gel in each panel. **a**, *mdr*1 expression in adjacent normal mucosa (N) and primary colon tumours (1°) from three patients, **b**, detection of *mdr*1 transcripts in normal (nl), tumour (1°) and lymph node metastasis (ly) from a single patient. From both panels a 4.5 kb *mdr*1 message is detected in seven of 11 liver metastases with an additional larger message detected in patient 1 **a**.

Levels of expression of *mdr*1 mRNA varied widely between tumour samples. Nevertheless, *mdr*1 mRNA was readily detected in 7/11 liver metastases although no *mdr*1 message was detected in the one lymph node metastasis that was evaluated. In two additional patients, we were able to obtain both the primary tumour and the corresponding liver metastasis. In one case, the primary tumour and the corresponding liver metastasis expressed comparable levels of *mdr*1 transcripts; in the second case *mdr*1 mRNA levels were much higher in the metastasis of the second patient (data not shown). When possible, tumour samples were run in parallel with adjacent normal mucosa obtained at the time of surgery. The relative level of *mdr*1 mRNA detected in primary tumours and adjacent normal tissues also varied considerably, with normal tissues expressing higher levels in two of the three pairs evaluated (Figure 1, panel a).

*Characterisation of MDR in human colon carcinoma cell lines*

Although every attempt has been made to standardise loaded samples in Northern blot analysis, heterogeneity within tumours and variability between tumour content make interpretations of results difficult. Immunohistochemistry and *in situ* hybridisation have been used previously to resolve this problem, however, such approaches cannot relate Pgp expression to functionality. To clarify this issues we have used a panel of colon carcinoma cell lines representing a more homogeneous starting cell population for the analysis of *mdr*1 mRNA/Pgp expression and Pgp function. Nineteen human colon carcinoma cell lines were classified according to differentiation state based on a variety of established criteria that include the histopathology of the original tumour and subsequent xenografts, carcinoembryonic antigen (CEA) production and, in some cases, invasive potential (Table I). In this way, the panel of human colon cell lines represents a range of differentiation phenotypes, *in vitro* and *in vivo*, with different invasive and metastatic potentials. From the data presented in Table I, histologically well differentiated cell lines exhibited many characteristics distinct from the phenotype expressed by histologically moderate or poorly differentiated cells.

**Table I** Characterisation of human colorectal cell panel

| *Cell line* | *Differentiation status*[a] | *CEA production*[b] *(ng/ml/10⁶ cells)* | *Invasion through matrigel*[c] |
|---|---|---|---|
| CX-1 | M/W | 35 | 31% |
| HTB-39 (HT29) | M/W | 7 | ND[d] |
| CCL238 (SW1417) | N.T.[e] | 24 | 38% |
| CL187 (LS180) | M/W | 525 | ND |
| CL188 (LS174T) | M/W | 5119 | ND |
| CCL229 (LoVo) | M/W | 882 | ND |
| HTB-37 (CaCo-2) | M/W | 27 | ND |
| CCL233 (SW1116) | M/W | 120 | ND |
| CCL-221 (DLD-1) | M/P | 1.2 | ND |
| Moser | M/P | <0.5 | ND |
| Clone D | M/P | <0.5 | ND |
| CCL235 (SW837) | M/P | 7 | 47% |
| CCL231 (SW48) | M/P | <0.5 | ND |
| CCL227 (SW620) | P | <0.5 | ND |
| CCL228 (SW 480) | P | 0.7 | 79% |
| Clone A | P | <0.5 | 100% |
| CCL222 | P | 2.5 | ND |
| CCL220.1 | P | <0.5 | ND |
| MIP 101 | P | <0.5 | 75% |

[a]Differentiation established from xenograft study. M/W = moderate/well; M/P = moderate/poor; P = poor. [b]CEA was determined on conditioned media using the Hoffman LaRoche radioimmunoassays. [c]Expressed as a percentage of Clone A values from Daneker *et al.* (1989). [d]ND = not determined. [e]NT = non-tumorigenic.

### *Expression of* mdr*1 mRNA and Pgp in colon cells*

Northern blot analysis of RNA from a representative panel of colon cell lines showed expression of a 4.5 kb *mdr*1 transcript in the majority of cell lines evaluated (12/19) (Figure 2). *Mdr*1 mRNA was detected in only 3/8 well differentiated cell lines, and in 9/11 of the moderate and poorly differentiated cell lines (Table II). Higher levels of *mdr*1 mRNA were apparent in the moderate and poorly differentiated cell lines with the highest levels found in Clone A and MIP 101, which were derived from, and are classified as, representative of poorly differentiated phenotypes (Dexter *et al.*, 1979, 1981; Niles *et al.*, 1987). The wide range of *mdr*1 mRNA levels detected in colon cell lines (Table II) was analogous to our observations with colon tumour tissue (Figure 1).

Pgp biosynthesis was established after overnight labelling of cells with $^{35}$S-methionine followed by immunoprecipitation with a polyclonal *mdr*1 antibody (Figure 3, Table II). In a number of cell lines small amounts of the 170 kd P-glycoprotein were detected where no *mdr*1 mRNA was detected in repeated Northern blot analysis (e.g. CaCo-2, Figure 3). Pgp was detected in 5/8 well differentiated and 9/10 moderate and poorly differentiated cell lines tested. In comparison to well differentiated colon cells, the moderate and poorly differentiated cell lines displayed higher levels of Pgp, proportional to the respective cellular levels of *mdr*1 mRNA detected (Table II). The highest levels of Pgp were found in the poorly differentiated Clone A and MIP 101 cell lines (Figure 3, top panel). The antibody used in these experiments immunoprecipitates a second protein of >200 kD. This protein appears unrelated to Pgp, since it is precipitated from cell lines which do not express detectable P-glycoprotein (HT29, CCL238). However, it serves as a useful internal control for verification of protein standardisation in all experiments.

**Figure 2** Northern blot analysis showing level of 4.5 kb *mdr*1 transcript in a representative panel of colon carcinoma cell lines.

Repetition of experiments following overnight labelling with $^{32}$P-orthophosphate confirmed previous observations of phosphorylation of the 170 kd Pgp (Figure 3, lower panel), where the degree of phosphorylation was found to be a good indicator of Pgp pump activity (Table II) suggesting that phosphorylation is a critical post-translational event in establishing Pgp function.

### *Altered Pgp expression in colon cell lines*

Previous work by other investigators have demonstrated that Pgp is synthesised as a 140 kd precursor which matures, via N-linked glycosylation, to a 170 kd membrane associated Pgp

**Figure 3** Immunoprecipitation of P-glycoprotein from a panel of colon carcinoma cell lines. Cells labelled for 18 h with $^{35}$S-methionine (upper panel) or $^{32}$P-orthophosphate (lower panel) prior to precipitation. In both experiments P-glycoprotein (Pgp) is resolved as the mature 170 kD form.

**Table II** Characterisation of MDR in representative panel of human colon carcinoma cell lines

| Cell lines | [³H]-Daun (nmoles/10⁶ cells)[a] – Ver | + Ver | (DMF)[c] | Dox $IC_{50}$ (μM)[b] – Ver | + Ver | (DMF)[c] | mdr1 mRNA/ Pgp biosynthesis[d] | Pgp surface expression[e] |
|---|---|---|---|---|---|---|---|---|
| *Moderate/Well* | | | | | | | | |
| CX-1 | 1.89 | 1.93 | (1.0) | 0.63 | 0.59 | (1.0) | 0/0 | |
| HT29 | 2.17 | 2.17 | (1.0) | 0.05 | 0.05 | (1.0) | 0/0 | – |
| CCL238 | 2.29 | 1.84 | (0.8) | ND[f] | ND | | 0/0 | |
| CL187 | 1.46 | 1.49 | (1.0) | ND | ND | | 0/+ | |
| CL188 | 2.24 | 2.66 | (1.2) | 0.28 | ND | | +/+ | + |
| LoVo | 1.53 | 1.25 | (0.8) | ND | ND | | +/+ | +/– |
| CaCo-2 | 2.34 | 2.42 | (1.0) | 1.67 | 1.67 | (1.0) | 0/+ | |
| CCL233 | 0.97 | 1.24 | (1.3) | ND | ND | | +/++ | |
| *Moderate/Poor* | | | | | | | | |
| DLD-1 | 1.58 | 1.94 | (1.3) | 0.37 | 0.33 | (1.1) | +++/+ | – |
| Moser | 0.72 | 1.83 | (2.5) | 1.10 | 0.34 | (3.0) | +++/+++ | ++++ |
| Clone D | 0.22 | 1.60 | (7.3) | ND | ND | | +++/+ | |
| CCL235 | 0.61 | 1.07 | (1.7) | ND | ND | | ++/++ | |
| CCL231 | 1.15 | 2.06 | (1.8) | 0.41 | 0.27 | (1.5) | +/+++ | ++ |
| *Poor* | | | | | | | | |
| CCL227 | 2.50 | 2.59 | (1.0) | 0.25 | 0.25 | (1.0) | 0/0 | – |
| CCL228 | 1.80 | 2.70 | (1.5) | 0.60 | 0.25 | (2.4) | ++/++ | |
| Clone A | 0.52 | 2.67 | (5.1) | 2.50 | 0.59 | (4.2) | ++++/++++ | ++++ |
| CCL222 | 1.99 | 2.34 | (1.2) | ND | ND | | 0/+ | |
| CCL220.1 | 1.33 | 0.98 | (0.8) | ND | ND | | +/ND | |
| MIP 101 | 0.42 | 1.84 | (4.4) | 1.55 | 0.32 | (4.8) | ++++/++++ | ++++ |

[a]Suspensions of colon cells ($2 \times 10^6$ ml$^{-1}$) were incubated for 90 min at 37°C with 10 μM [³H]daunorubicin (sp. act. 1 miCi/10 μM) in RPMI 1640 medium supplemented with 10% FCS, 2 mM glutamine and 25 mM HEPES (pH 7.25) with glucose concentration of 11 mM. Verapamil (10 μg ml$^{-1}$) was added immediately before daunorubicin and incubations were terminated by layering 0.5 ml cells over silicone and microcentrifuging at 12,000 *g* for 1 min. Pellets were dissolved in NaOH and counted by liquid scintillation spectrometry. Values are the means of two determinations. [b]$IC_{50}$ values were determined using the SRB assay described in Methods. Verapamil (10 μg ml$^{-1}$) was added immediately before doxorubicin. Cells were treated for 3 h and SRB optical density determined on trichloroacetic acid (10%) fixed cells, 4 days after drug treatment. Values are the means of no less than three experiments. [c]Dose modifying factors (DMF) were calculated as follows:

$$\text{Drug Accumulation; } \frac{\text{nmoles daun}/10^6 \text{ cells + verapamil}}{\text{nmoles daun}/10^6 \text{ cells control}} \quad \text{DOX } IC_{50}; \ \frac{IC_{50} \text{ control}}{IC_{50} + \text{verapamil}}$$

[d]*mdr*1 mRNA levels were estimated by Northern blot analysis, and are presented relative to the level expressed in MIP 101; 0, no *mdr*1 mRNA detected; + + + +, equivalent to MIP 101. Pgp biosynthesis was calculated by labelling cells for 16 h with ³⁵S-methionine followed by immunoprecipitation with excess affinity purified *mdr*1 antibody. The immune complexes were separated in a 7.5% SDS-PAGE. The values shown are 0; no Pgp detected in repeated experiments, + + + + equivalent to MIP 101. [e]Surface expression determined using Mab 4E3.16 binding of live cells assessed in FACS analysis. [f]ND = not determined.

(Greenberger *et al.*, 1988; Richert *et al.*, 1988). Labelling of cells for shorter periods (3 h) with $^{35}$S-methionine facilitates resolution of both the immature and mature forms of the P-glycoprotein (Figure 4, upper panel). Immunoprecipitation of Pgp from cell lysates of CCL 228, Clone A and MIP 101 (Figure 4, upper panel) show both the 140 kd precursor and 170 kd mature Pgp, whilst the Moser cell line displays a faster migrating mature P-glycoprotein (Figure 4). Since the precursor 140 kD molecule of Moser migrates to the same level as that observed in other lines expressing MDR, it is likely that the altered mature P-glycoprotein in this cell line results from underglycosylation. It is interesting to note that Moser displays significant Pgp pump activity despite aberrant processing of the immature form (Table II). Representation of the 140 kd precursor in DLD-1 is at comparable levels to that of Moser and yet only minimal mature P-glycoprotein is resolved in immunoprecipitation experiments (compare lanes, Figure 4, upper panel). The difference in migration of the 170 kd Pgp in Moser and its under representation in DLD-1 is best resolved in experiments where cells were labelled for a 3 h period with $^{32}$P-orthophosphate. Immunoprecipitation of phosphorylated Pgp in the same panel of cell lines (Figure 4, lower panel) reveals minimal and aberrant signals in DLD-1 and Moser respectively. Most notable in these assays is the resolution of a single 170 kD protein corresponding to the mature 170 kD P-glycoprotein. From these results we conclude only the mature P-glycoprotein is phosphorylated in constitutively expressed MDR.

### *Detection of cell surface associated Pgp in colon cell lines*

One explanation for the different MDR phenotypes displayed by Moser and DLD-1 in the presence of comparable levels of P-glycoprotein biosynthesis, is that the reduction in mature (170 kD) Pgp observed in DLD-1 results in the absence of membrane associated product. To address this possibility we have used a monoclonal antibody (4E3.16) recognising an external epitope of Pgp, capable of binding P-glycoprotein on live cells. Incubation of a representative panel of colon cell lines with the fluorochrome tagged antibody, followed by fluorescence activated cell sorting demonstrates the level of expression of surface associated Pgp (Figure 5). In this study DLD-1 showed minimal levels of detectable surface Pgp (Figure 5, panel b) contrasting with the Moser cell line which displayed high levels of surface staining (Figure 5, lane f), comparable to that recorded in the most drug resistant colon cell lines Clone A and MIP 101 (Figure 5, panels g and h respectively). Comparative levels of surface Pgp within the cell panel, directly correlated with the MDR phenotype established in drug uptake assays (Table II).

### *Functional assessment of MDR in colon cells*

Colon cells expressed a range of differences with respect to net daunorubicin accumulation and doxorubicin cytotoxicity (Table II). Colon carcinoma cells accumulated between 0.42 nmoles daunorubicin/10$^6$ cells to 2.5 nmoles dauno-

**Figure 4** Immunoprecipitation of P-glycoprotein from a representative panel of colon carcinoma cell lines following a short labelling period (3 h) with $^{35}S$-methionine (upper panel) or $^{32}P$-orthophosphate (lower panel). P-glycoprotein is resolved as a doublet (upper panel) representing the mature 170 kD glycoprotein and the immature unglycosylated 140 kD precursor. Note the faster migrating mature P-glycoprotein associated with Moser (unlabelled arrow) and the absence of detectable mature P-glycoprotein (170) in DLD-1 (upper panel). Parallel experiments with immunoprecipitation of Pgp from $^{32}P$-orthophosphate cell lysates results in detection of the mature 170 kD P-glycoprotein (lower panel), once again demonstrating altered migration in the Moser cell line.

rubicin/$10^6$ cells, with the lowest levels being found in *mdr*1 mRNA/Pgp positive colon cells lines. The doxorubicin concentration that inhibited colon cell growth by 50% ($IC_{50}$; 3 h drug exposure) varied as much as 50-fold among the various colon cell lines, ranging from 0.05 μM to 2.5 μM, with *mdr*1 mRNA/Pgp-positive cells having the highest $IC_{50}$ values.

Pgp function was estimated by determining the fold increase in net [$^3$H]-daunorubicin accumulation and doxorubicin cytotoxicity (decreases in $IC_{50}$ value) that was caused by treating cells with an antagonist of Pgp (i.e. verapamil, 25 μM) (Table II). Verapamil treatment increased drug accumulation in colon cell lines by 20 to 510%, and resulted in a corresponding increase in doxorubicin cytotoxicity (i.e. fold decrease in $IC_{50}$ value). The percentage increase in drug accumulation and cytotoxicity was used to estimate Pgp function, and was found to correlate with *mdr*1 mRNA/Pgp expression levels. For example, colon cell lines that had no measurable *mdr*1 mRNA or Pgp (e.g. CCL238, CX-1, HT29, CCL227), exhibited no functional Pgp activity as determined by the verapamil- inducible drug uptake and cytotoxicity assays (Table II). Cells expressing the lowest detectable levels of *mdr*1 mRNA/Pgp (e.g. LoVo, CL187, CaCo-2, CCL220.1) also had no measurable increases in drug accumulation or cytotoxicity in response to verapamil. However, cell lines expressing low to moderate levels of *mdr*1 mRNA/Pgp (e.g. CL188, CCL231, DLD-1), did exhibit a range (10–100%) of verapamil-induced increases in drug accumulation and cytotoxicity. The highest levels of Pgp activity (>250%) were found in those cell lines expressing the highest levels of *mdr*1 mRNA/Pgp (i.e. Moser, MIP 101, Clone A). Only 2/8 of the well differentiated cell lines expressed the MDR phenotype as defined by these criteria, and these well differentiated MDR positive cell lines (i.e. CL188 and CCL233) exhibited the lowest measurable levels of functional activity (20–30%). In contrast, 9/11 moderate and poorly differentiated cell lines displayed functional MDR phenotypes exhibiting Pgp activities ranging from 20–>500%.

*Non-Pgp mechanisms of MDR in colon carcinoma cells*

Representative colon cell lines were analysed for cross-resistance and chemosensitivity profiles against a battery of chemotherapeutic drugs (Table III). In these studies, the cells were exposed to drugs for 48 h. Cells expressing high constitutive levels of *mdr*1 mRNA/Pgp i.e. MIP 101, Clone A, Moser, were cross-resistant to drugs normally associated with the MDR phenotype e.g. vincristine, etoposide and doxorubicin. The relative degree of drug resistance was determined by comparing the $IC_{50}$ values in these cells to a representative Pgp-negative cell line e.g. CCL238. The drug sensitivity profile of CCL 238 was not appreciably different to the sensitive human leukaemia cell line HL60. The relative resistance (i.e. $IC_{50}$ of MDR+ cells/$IC_{50}$ CCL238) of these high expressing cell lines was proportional to the cellular levels of *mdr*1 mRNA/Pgp and ranged from 2.5 to 50-fold for doxorubicin 11 to 50-fold for vincristine and 5 to 10-fold for etoposide. While, vincristine resistance correlated with the level of *mdr*1 mRNA/Pgp expression, a direct relationship between etoposide resistance and *mdr*1 mRNA/Pgp expression could not be established. For example, CCL228 expressed lower levels of *mdr*1 mRNA/Pgp compared to MIP 101, Clone A or Moser (Table III) and were proportionally less resistant to vincristine, and yet of the MDR positive cells, CCL228 was the most resistant to etoposide. Moreover, the cell lines demonstrating the highest levels of etoposide resitance i.e. CaCo-2 and CX-1 were not MDR-positive as shown by the lack of *mdr*1 expression as well as by functional assays. As might be expected the MDR cells were not cross-resistant to 5-fluorouracil, cisplatin or chlorambucil (Table III). Cells exhibiting a modest degree of functional Pgp activity (Table II) expressed detectable but correspondingly lower levels of *mdr*1 mRNA/Pgp. These cells, CL188, DLD-1, CCL231, were not cross-resistant to the standard MDR drugs. For example, both *mdr*1 mRNA and Pgp were detected in CCL231 cells at levels that were proportional to the verapamil mediated increase in 3H-daunorubicin accumulation of 80% (Table II). However, CCL231 was equally sensitive to doxorobucin, vincristine and etoposide compared to our non-Pgp reference CCL238 colon carcinoma cell line. These observations suggest that in colon cancer, other mechanisms of resistance e.g., altered topoisomerase, glutathione may also contribute to the pattern of resistance particularly when *mdr*1 mRNA/Pgp when expressed at low levels. However at higher levels of expression, *mdr*1 mRNA/Pgp becomes the major determinant of resistance.

### Discussion

Multidrug resistance has been implicated as a contributing factor in the intrinsic resistance of several solid tumours, including colon, ever since the original observation by Thiebaut *et al.* (1987) that the *mdr*1 gene product was constitutively expressed in the normal tissues from which these tumours were derived. Subsequent studies by Fojo *et al.* (1987*b*), Goldstein *et al.* (1989) and others, provided additional support for this by demonstrating that *mdr*1 mRNA was often present in the primary colon tumour specimens before patients received chemotherapy. A recent report by Weinstein *et al.* (1991) demonstrated that immunodetectable Pgp was found in a high percentage of invasive carcinoma

**Figure 5** Surface expression of P-glycoprotein in colon carcinoma cell lines using monoclonal antibody 4E3.16 in FACS analysis. **a**, HT29; **b**, DLD-1; **c**, CCL 229; **d** CL 188; **e**, CCL-231; **f**, Moser; **g**, Clone A; **h**, MIP 101. Unshaded area in each panel represents surface Pgp detected on live cells with Mab 4E3.16. Shaded area in each panel represents analysis with class matched control antibody IgG2a.

cells and in the lymph node metastasis of patients with colon cancer. The present study extends these observations by demonstrating that *mdr*1 mRNA was also expressed in liver metastasis. These findings demonstrate that colon carcinoma cells not only retain the capacity to express the *mdr*1 gene, but that this characteristic of the normal colonic epithelium can be maintained throughout all stages of colon tumour progression. This observation is consistent with a recent study in neuroblastoma patients in which Pgp was detected in a high percentage of the advanced lesions (Chan *et al.*, 1991). The findings in neuroblastoma and colon cancer patients provide a compelling basis for understanding why metastatic disease is refractory to certain chemotherapeutic drugs, particularly if the metastasis developed from primary tumours that were derived from Pgp-expressing normal tissues.

In this study, *mdr*1 mRNA levels varied considerably in colon tumours and in normal tissue, and is consistent with all previous studies evaluating surgical material (Fojo *et al.*, 1987*a*,*b*; Lai *et al.*, 1989; Goldstein *et al.*, 1989). A relationship between *mdr*1 mRNA/Pgp expression and clinical drug resistance is particularly hard to establish in colon cancer because these patients rarely receive chemotherapy with MDR-associated drugs. Therefore, one cannot relate expression levels with patient outcome in response to chemotherapy as has been done in clinical studies involving patients with soft tissue sarcomas (Gerlach *et al.*, 1987), myelomas (Dalton *et al.*, 1989), or neuroblastomas (Chan *et al.*, 1991). Previous studies using surgical material from untreated colon cancer patients have attempted to place significance on expression levels in tumours that were higher than the levels expressed in adjacent normal tissues. However, normal tissue *mdr*1

**Table III** Chemosensitivity of human colorectal cell lines

| Cell line | $IC_{50}$ Values ($\mu g\ ml^{-1}$)[a] DOX[b] | VIN | ETO | 5-FU | CDDP | CHLR |
|---|---|---|---|---|---|---|
| *Well/moderately differentiated* | | | | | | |
| CCL238 | 0.12 | 0.02 | 1.1 | 8.6 | 1.0 | 4 |
| CaCo-2 | 2.50 | 0.05 | 42.0 | 9.0 | 5.4 | >100 |
| CX-1 | 0.45 | 0.005 | 16.7 | 0.2 | 6.0 | >100 |
| HT-29 | 0.22 | 0.003 | 7.8 | 0.6 | 3.9 | >100 |
| LoVo | 0.24 | 0.05 | 1.5 | 0.3 | 3.0 | 2 |
| CL188 | 0.04 | 0.03 | 0.7 | 2.8 | 2.0 | ND |
| *Moderate/poorly differentiated* | | | | | | |
| CCL231 | 0.21 | 0.02 | 1.4 | 4.7 | 2.7 | 4 |
| DLD-1 | 0.36 | 0.04 | 1.8 | 1.7 | 7.6 | 9 |
| Moser | 5.00 | 0.36 | 5.4 | 14 | 5.4 | 56 |
| *Poorly differentiated* | | | | | | |
| CCL227 | 0.05 | 0.002 | 1.0 | 16 | 2.1 | 6 |
| CCL228 | 0.30 | 0.22 | 9.9 | 9.8 | 2.1 | >100 |
| Clone A | 3.0 | 0.80 | 8.7 | 5.4 | 5.4 | 85 |
| MIP 101 | 4.5 | 1.00 | 5.5 | 8.5 | 4.3 | 85 |
| *Leukaemia* | | | | | | |
| HL60 | 0.19 | ND | 2.1 | 6.3 | 5.7 | 24 |

[a]$IC_{50}$ values were determined using the SRB assay, and were calculated using the formula $T\text{-}T_0/C\text{-}T_0$, as described in Methods. In all experiments, log phase cells ($1-2 \times 10^4$ cells per well in 96-well microtitre plates) were treated with chemotherapeutic drugs for 48 h. Values are the means of duplicate wells, and are representative of multiple experiments. [b]Abbreviations used are DOX = doxorubicin; VIN = vincristine; ETO = etoposide; 5-FU = 5-fluorouracil; CDDP = cisplatin; and CHLR = chlorambucil.

mRNA levels were frequently higher than tumour levels (Fojo *et al.*, 1987*b*). It has never actually been established if normal colon cells are also drug resistant, and at what level of constitutive Pgp expression does resistance actually occur. This may be important given that most of what we know about Pgp and resistance has come from studies with cell lines that were selected on the basis of functional criteria (i.e. they survived treatment with escalating doses of chemotherapy). Thus we felt that human colon tumour cells offered the best available model to study the relationship between constitutive *mdr*1 mRNA/Pgp expression and functional resistance, and the possible relationship between *mdr*1 mRNA/Pgp expression and colon tumour progression. Cell lines DLD-1, Clone A, Clone D, MIP 101, and Moser, all of which express P-glycoprotein, were established from tumour material prior to exposure from chemotherapeutic agents.

The colon carcinoma cell lines used in this study were selected primarily on the basis of differentiation using established criteria e.g. histology, carcinoembryonic antigen secretion, these parameters are summarised in Table I. Previous studies have shown that poorly differentiated colon carcinoma cells were the most aggressive as assessed using *in vitro* adhesion and invasion assays (Daneker *et al.*, 1989). These *in vitro* studies support clinical observations which have related poorly differentiated colon tumour histologies with a poor prognosis and a greater likelihood of metastatic involvement. It is clear from the biochemical data presented that the most aggressive, poorly differentiated colon tumour cell lines within the panel expressed the highest constitutive levels of P-glycoprotein correlating with their relative functionality recorded in drug uptake assays. In contrast to previous reports (Mickley *et al.*, 1989; Mizoguchi *et al.*, 1990) we find no correlation between differentiation status and the MDR phenotype in the cell panel studied, but observe that P-glycoprotein expression can be maintained in both well and poorly differentiated colon cell lines. This observation is consistent with that reported by Park *et al.* (1990). Although it can be argued that cell lines are not representative of the *in vivo* lesion, it should be considered that, since poorly differentiated tumours represent approximately 5% of the colorectal tumours resected, it is difficult to generate sufficient numbers to definitively evaluate the differentiation/MDR phenotype association using human tumour material. Interestingly, Pgp was immunoprecipitated from some cell lines where no MDR functionality was detected. This may reflect the limitations of sensitivity of the assays used or the requirement for a threshold level of Pgp expression to acquire functional drug resistance. However, moderate/well differentiated colon cell lines HT29 and CCL238 consistently displayed an absence of detectable *mdr*1 message or immunoprecipitable Pgp and demonstrated a lack of MDR functionality in repeated assays consistent with results reported by Spoelstra *et al.* (1991). The amount of mature 170 kD P-glycoprotein resolved in protein standardised immunoprecipitation was directly reflected by the phosphorylation status of Pgp, revealing an absolute correlation with MDR functionality within the colon carcinoma cell panel studied.

Previous characterisation of the biosynthesis of Pgp has identified a 140 kD precursor molecule which is processed, via N-linked glycosylation, to a 170 kD species identified as the mature P-glycoprotein (Greenberger *et al.*, 1988; Richert *et al.*, 1988). Despite the altered maturation of Pgp in the Moser cell line the mature form is found to be phosphorylated and cell surface associated establishing the MDR phenotype displayed by Moser in functional assays. In contrast, DLD-1 displayed only minimal detectable functional Pgp activity despite relatively high expression levels of *mdr*1 mRNA in Northern blot analysis. Consistent with this observation is the lack of mature Pgp (170 kD) found in this cell line despite detection of comparable levels of the precursor molecule (140 kD) to that found in other cell lines within the panel e.g. Moser. This apparent lack of processing of the DLD-1 Pgp is further reflected in the absence of phosphorylated Pgp and the lack of P-glycoprotein at the cell surface. The correlation observed between the phosphorylation status and membrane association of Pgp with the MDR phenotype implicates both of these factors as important in establishing cellular drug resistance. In all *mdr*1 immunoprecipitation protocols involving $^{32}P$-orthophosphate labelling, Pgp was resolved as a single band comigrating with the mature 170 kD P-glycoprotein. From these results we conclude that only the mature form of P-glycoprotein is phosphorylated in colon cells.

The functional significance of Pgp processing may be particularly important in non-selected, constitutively expressing cells and tumours. This possibility further complicates attempts to attribute the clinical resistance of colon cancer solely to changes in the expression of *mdr*1 mRNA and/or detection of Pgp in immunohistochemistry or immunoprecipitation protocols. Although our results, showing a high frequency of *mdr*1 mRNA/Pgp expression in colon tumour specimens and colon carcinoma cell lines supports a role for MDR in the clinical resistance of colon carcinomas, we also report that the low constitutive levels of Pgp expressed in many colon carcinoma cells may not be sufficient to confer resistance. Moreover, *mdr*1 mRNA/Pgp expression levels correlated poorly with etoposide resistance, and several colon carcinoma cell lines with appreciable levels of expression and demonstrated Pgp activity (e.g. CCL 231) were no more resistant to MDR drugs (e.g. vincristine) than were Pgp-negative colon cell lines. These observations are consistent with the view that multiple mechanisms in addition to Pgp (e.g. topoisomerase, 6-alkylguanine-DNA-alkyltransferase, glutathione peroxidase) contribute to the overall resistance of colorectal cancer (Kramer *et al.*, 1988; Redmond *et al.*, 1991).

This work was supported by NIH grants CA50473 (R.K.); CA42944 and CA44704 (I.C.S.). We are grateful to Carol Ann Hannan for typing the manuscript.

### References

ARCECI, R.J., STIEGLITZ, K., BRAS, J., SCHINKEL, A., BAAS, F. & CROOP, J. (1993). A monoclonal antibody to an external epitope of the human MDR1 P-glycoprotein. *Cancer Res.* (in press).

BECH-HANSEN, N.T., TILL, J.E. & LING, V. (1986). Pleiotropic phenotype of colchicine-resistant CHO cells: cross-resistance and collateral sensitivity. *J. Cell Physiol.*, **88**, 23–32.

CHAN, H.S.L., HADDED, G., THORNER, D.S., DEBOER, G., LIN, Y.P., ONDRUSEK, N., YEGERH, ?. & LING, V. (1991). P-glycoprotein expression as a predictor of the outcome of therapy for neuroblastoma. *New Engl. J. Med.*, **325**, 1608–1614.

CHEN, C.J., CHIN, J.E., UEDA, K., CLARK, D.P., PASTAN, I., GOTTESMAN, M.M. & RONINSON, I.B. (1986). Internal duplication and homology with bacterial transport proteins in the mdr 1 (P-glycoprotein) gene from multidrug-resistant human cells. *Cell*, **47**, 381–389.

CHIRGWIN, J.W., PRZYBYLA, A.E., MACDONALD, R.J. & RUTTER, W.J. (1979). Isolation of biologically active ribonucleic acid from sources enriched in ribonuclease. *Biochemistry*, **18**, 5294–5299.

CORDON-CARDO, C., O'BRIEN, J.P., CASALS, D., RITTMAN-GRAUER, L., BIEDLER, J.L., MELAMED, M.R. & BERTINO, J.R. (1989). Multidrug-resistance gene (P-glycoprotein) is expressed by endothelial cells at blood-brain barrier sites. *Proc. Natl Acad. Sci. USA*, **86**, 695–698.

CORDON-CARDO, C., O'BRIEN, J.P., BOCCIA, J., CASALS, D., BERTINO, J.R. & MELAMED, M.R. (1990). Expression of the multidrug resistance gene product (P-glycoprotein) in human normal and tumor tissues. *J. Histochem. Cytochem.*, **38**, 1277–1287.

CORNWELL, M.M., GOTTESMAN, M.M. & PASTAN, I.H. (1986). Increased vinblastine binding to membrane vesicles from multidrug-resistant KB cells. *J. Biol. Chem.*, **261**, 7921–7928.

DALTON, W.S., GROGAN, T.M., MELTZER, P.S., SCHEPER, R.J., DURIE, B.G.M., TAYLOR, G.W., MILLER, T.P. & SALMON, S.E. (1989). Drug-resistance in multiple myeloma and non-Hodgkin's lymphoma: detection of P-glycoprotein and potential circumvention by addition of verapamil to chemotherapy. *J. Clin. Oncol.*, **17**, 415–424.

DANEKER, Jr, G.W., PIAZZA, A.J., STEELE, Jr, G.D. & MERCURIO, A.M. (1989). Relationship between extracellular matrix interactions and degree of differentiation in human colon carcinoma cell lines. *Cancer Res.*, **49**, 681–686.

DEXTER, D.L., BARBOSA, J.A. & CALABRESI, P. (1979). N,N-Dimethylformamide-induced alteration of cell culture characteristics and loss of tumorigenicity in cultured human colon carcinoma cells. *Cancer Res.*, **39**, 1020–1025.

DEXTER, D.L., SPREMULLI, E.N., FLIGIEL, Z., BARBOSA, J.A., VOGEL, R., VANVOORHEES, A. & CALABRESI, P. (1981). Heterogeneity of cancer cells from a single human colon carcinoma. *Am. J. Med.*, **71**, 949–956.

FOJO, A.T., SHEN, D.W., MICKLEY, L.A., PASTAN, I. & GOTTESMAN, M.M. (1987*a*). Intrinsic drug resistance in human kidney cancer is associated with expression of a human multidrug-resistance gene. *J. Clinical Oncol.*, **5**, 1922–1927.

FOJO, A.T., UEDA, K., SLAMON, D.J., POPLACK, D.G., GOTTESMAN, M.M. & PASTAN, I. (1987*b*). Expression of a multidrug-resistance gene in human tumors and tissues. *Proc. Natl Acad. Sci. USA*, **84**, 265–269.

GERLACH, J.H., BELL, D.R., KARAKOUSIS, C., SLOCUM, H.K., KARTNER, N., RUSTUM, Y.M., LING, V. & BAKER, R.M. (1987). P-glycoprotein in human sarcoma: evidence for multidrug resistance. *J. Clin. Oncol.*, **5**, 1452–1460.

GOLDSTEIN, L.J., GALSKI, H., FOJO, A., WILLINGHAM, M., SHINN-LIANG, L., GAZDAR, A., PIRKER, R., GREEN, A., CRIST, W., BRODEUR, G.M., LIEBER, M., COSSMAN, J., GOTTESMAN, M.M. & PASTAN, I. (1989). Expression of a multidrug resistance gene in human cancers. *J. Natl Cancer Inst.*, **81**, 116–124.

GOLDSTEIN, L.J., FOJO, A.T., UEDA, K., CRIST, W., GREEN, A., BRODEUR, G., PASTAN, I. & GOTTESMAN, M.M. (1990). Expression of the multidrug resistance, MDR1, gene in neuroblastomas. *J. Clin. Oncol.*, **8**, 128–136.

GREENBERGER, L.M., WILLIAMS, S.S., GEORGES, E., LING, V. & HOROWITZ, S.B. (1988). Electrophoretic analysis of P-glycoproteins produced by mouse J774-2 and chinese hamster ovary multidrug-resistant cells. *J. Natl Cancer Inst.*, **89**, 506–510.

HALLER, D.G. (1988). Chemotherapy in gastrointestinal malignancies. *Sem. Onc.*, **15** (No 3 suppl 4): 50–64.

KARTNER, N., RIORDAN, J.R. & LING, V. (1983). Cell surface P-glycoprotein associated with multidrug resistance in mammalian cell lines. *Science*, **221**, 1285–1288.

KRAMER, R.A., ZAKHER, J. & KIM, G. (1988). Role of the glutathione redox cycle in acquired and *de novo* multidrug resistance. *Science*, **241**, 694–698.

KRAMER, R.A. (1989). Multidrug resistance in cancer cells: biochemical mechanisms. *Gastroenterology*, **96**, 1214–1215.

LAI, S.L., GOLDSTEIN, L.J., GOTTESMAN, M.D., PASTAN, I., TSAI, C.-M., JOHNSON, B.E., MULSHINE, J.L., IHDE, D.C., KAYSER, K. & GAZDAR, A.F. (1989). MDR1 gene expression in lung cancer. *J. Natl Cancer Inst.*, **81**, 1144–1150.

MA, D.D.F., SCURR, R.D., DAVEY, R.A., MACKERTICH, S.M., HARMAN, D.H., DOWDEN, G., ISBISTER, J.P. & BELL, D.R. (1987). Detection of a multidrug resistant phenotype in acute non-lymphoblastic leukemia. *Lancet*, **1**, 135–137.

MIZOGUCHI, T., YAMADA, K., FURUKAWA, T., HIDAKA, K., HISATSUGU, T., SHIMAZU, H., TSURUO, T., SUMIZAWA, T. & AKIYAMA, S.-I. (1990). Expression of the MDR1 gene in human gastric and colorectal carcinomas. *J. Natl Cancer Inst.*, **82**, 1679–1683.

MONKS, A., SCUDIERO, D., SKEHAN, P., SHOEMAKER, R., PAULL, K., VISTICA, D., HOSE, C., LANGLEY, J., CRONISE, P., VAIGRO-WOLFF, A., GRAY-GOODRICH, M., CAMPBELL, H., MAYO, J. & BOYD, M. (1991). Feasibility of a high-flux anticancer drug screen using a diverse panel of cultured human tumor cell lines. *J. Natl Cancer Inst.*, **83**, 757–766.

NILES, R.M., WILHELM, S.A., STEELE Jr, G.D., BURKE, B., CHRISTENSEN, T., DEXTER, D., O'BRIEN, J.M., THOMAS, P. & ZAMCHECK, N. (1987). Isolation and characterization of an undifferentiated human colon carcinoma cell line (MIP 101). *Cancer Investigation*, **5**, 545–552.

PARK, J.-G., KRAMER, B.S., LAI, S.-L., GOLDSTEIN, L.J. & GAZDAR, A.F. (1990). Chemosensitivity patterns and expression of human multidrug resistance-associated MDR1 gene by human gastric and colorectal carcinoma cell lines. *J. Natl Cancer Inst.*, **82**, 193–198.

REDMOND, S.M.S., JONCOURT, F., BUSER, K., ZIEMIECKI, A., ALTERMATT, H.J., FEY, M., MARGISON, G. & CERNY, T. (1991). Assessment of P-glycoprotein, glutathione-based detoxifying enzymes and $O^6$-alkylguanine-DNA alkyltransferase as potential indicators of constitutive drug resistance in human colorectal tumors. *Cancer Res.*, **51**, 2092–2097.

RICHERT, N.D., ALDWIN, L., NITECKI, D., GOTTESMAN, M.M. & PASTAN, I. (1988). Stability and covalent modification of P-glycoprotein in multidrug-resistant KB cells. *Biochemistry*, **27**, 7607–7613.

RIEHM, H. & BIEDLER, J.L. (1971). Cellular resistance to daunomycin in chinese hamster cells *in vitro*. *Cancer Res.*, **31**, 409–412.

RIOU, G.F., ZHOU, D., AHOMADEGBE, J.-C., GABILLOT, M., DUVILLARD, P. & LHOMME, C. (1990). Expression of multidrug-resistance (MDR1) gene in normal epithelia and in invasive carcinoma of the uterine cervix. *J. Natl Cancer Inst.*, **82**, 1493–1496.

SCHLAIFER, D., LAURENT, G., CHITTAL, S., TSURUO, T., SOUES, S., MULLER, C., CHARCOSSET, J.Y., ALARD, C., BROUSEET, P., MAZERRAOLLES, C. & DELSOL, G. (1990). Immunohistochemical detection of multidrug resistance associated P-glycoprotein in tumor and stromal cells in human cancers. *Br. J. Cancer*, **62**, 177–182.

SILVERBERG, E. & LUBERA, J. (1986). *A Cancer Journal for Clinicians*, **36**, 9–25.

SKEHAN, P., STORENG, R., SCUDIERO, D., MONKS, A., MCMAHON, J., VISTICA, D., WARREN, J.T., BOKESCH, H., KENNEY, S. & BOYD, M.R. (1990). New colorimetric cytotoxicity assay for anticancer-drug screening. *J. Natl Cancer Inst.*, **82**, 1107–1112.

SPOELSTRA, E.C., DEKKER, H., SCHUURHUIS, G.J., BROXTERMAN, H.J. & LANKELMA, J. (1991). P-glycoprotein drug efflux pump involved in the mechanisms of intrinsic drug resistance in various colon cancer cell lines. *Biochem. Pharm.*, **41**, 349–359.

SPOELSTRA, E.C., WESTERHOFF, H.V., DEKKER, H. & LANKELMA, J. (1992). Kinetics of daunorubicin transport by P-glycoprotein of intact cancer cells. *Eur. J. Biochem.*, **207**, 567–579.

THIEBAUT, F., TSURUO, T., HAMADA, H., GOTTESMAN, M.M., PASTAN, I. & WILLINGHAM, M.C. (1987). Cellular localization of the multidrug-resistance gene product P-glycoprotein in normal human tissues. *Proc. Natl Acad. Sci. USA*, **84**, 7735–7738.

UEDA, K., CLARK, D.P., CHEN, C.J., RONINSON, I.B., GOTTESMAN, M.M. & PASTAN, I. (1987). The human multidrug resistance (mdr1) genes. *J. Biol. Chem.*, **262**, 505–508.

WEINSTEIN, R.S., JAKATE, S.M., DOMINGUEZ, J.M., LEBOVITZ, M.D., KOUKOULIS, G.K., KUSZAK, J.R., KLUSENS, L.F., GROGAN, T.M., SACLARIDES, T.J., RONINSON, I.B. & COON, J.S. (1991). Relationship of the expression of the multidrug resistance gene product (P-glycoprotein) in human colon carcinoma to local tumor aggressiveness and lymph node metastasis. *Cancer Res.*, **51,** 2720–2726.

YANG, C.P.H., DEPINHO, S.G., GREENBERGER, L.M., ARCECI, R.J. & HOROWITZ, S.B. (1989). Progesterone interacts with P-glycoprotein in multidrug-resistant cells and in the endometrium of gravid uterus. *J. Biol. Chem.*, **264,** 782–788.