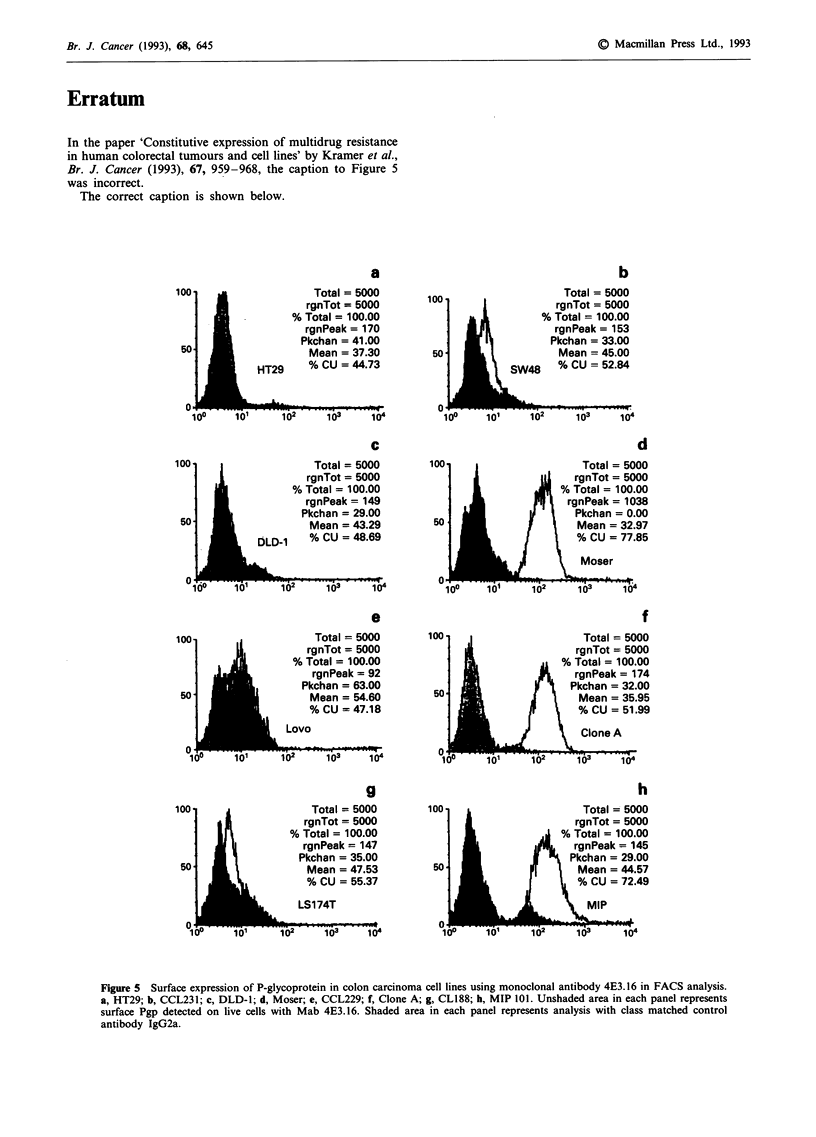# Erratum

**Published:** 1993-09

**Authors:** 


					
Br. J. Cancer (1993), 68, 645                                                                         ?   Macmillan Press Ltd., 1993

Erratum

In the paper 'Constitutive expression of multidrug resistance
in human colorectal tumours and cell lines' by Kramer et al.,
Br. J. Cancer (1993), 67, 959-968, the caption to Figure 5
was incorrect.

The correct caption is shown below.

a

HT29

Total = 5000
rgnTot = 5000
% Total = 100.00

rgnPeak = 170
Pkchan = 41.00
Mean = 37.30
% CU = 44.73

102      1i03        104

C

I

DLD-1

Total = 5000
rgnTot = 5000
% Total = 100.00

rgnPeak = 149
Pkchan = 29.00
Mean = 43.29
% CU = 48.69

100-

50

&  y l SW48

b

Total = 5000
rgnTot = 5000
% Total = 100.00

rgnPeak= 153
Pkchan = 33.00
Mean = 45.00
% CU = 52.84

*0   mumming  - 2  103  104. .
100  101  102       10. 4

d

100
50

I~        .    { I   -      1b

10o2        1603 -        0o4

e

Total = 5000
rgnTot = 5000
% Total = 100.00

rgnPeak = 92
Pkchan = 63.00
Mean = 54.60
% CU = 47.18

Lovo

102     103   104

g
Total = 5000
rgnTot = 5000
% Total = 100.00

rgnPeak = 147
Pkchan = 35.00
Mean = 47.53
% CU = 55.37
LS1 74T

o2     13     104

io4

f

100

50

h

100

50

0

Figure 5 Surface expression of P-glycoprotein in colon carcinoma cell lines using monoclonal antibody 4E3.16 in FACS analysis.
a, HT29; b, CCL231; c, DLD-l; d, Moser; e, CCL229; f, Clone A; g, CL188; h, MIP 101. Unshaded area in each panel represents
surface Pgp detected on live cells with Mab 4E3.16. Shaded area in each panel represents analysis with class matched control
antibody IgG2a.

100

50

0

1o

100

50-

II.

100
50

0

1b

100
50

IA

Br. J. Cancer (1993), 68, 645

v Macmillan Press Ltd., 1993

a